# PReS-FINAL-2017: Systemic onset juvenile idiopathic arthritis (SOJIA)-a 5-year survey in a pediatric rheumatology department

**DOI:** 10.1186/1546-0096-11-S2-P30

**Published:** 2013-12-05

**Authors:** A Čengić, A Omerčahić Dizdarević, V Selmanović, S Mesihović Dinarević

**Affiliations:** 1 Pediatric Alergoimmunorheumatology, Clinical Centre University of Sarajevo, Sarajevo, Bosnia and Herzegovina; 2 Pediatric Cardiology, Pediatric Clinic, Clinical Centre University of Sarajevo, Sarajevo, Bosnia and Herzegovina

## Introduction

Systemic onset Juvenile Idiopathic Arthritis (SoJIA) is rare pediatric disease, it accounts for 10% of children with Juvenile Idiopathic Arthritis. The onset of disese can be vary nonspecific and may suggest bacterial or viral infection, malignancy or other rheumatic disease. It is highly characterised by its extra-articular systemic illness features and, in some ways, it resembles a fever of unknown origin. Diagnosis is mostly clinical by using ILAR criteria (International League of Associations for Rheumatology).

## Objectives

To assess the frequency of presenting symptoms and laboratory findings in SoJIA patients over 5 years period.

## Methods

This is retrospective study on all SoJIA patients diagnosed in department of pediatric rheumatology from January 2008 until January 2013 using ILAR criteria. The medical records were reviewed. In each case age, gender, presenting symptoms and laboratory data were reviewed.

## Results

During the study period 12 patients were diagnosed, mean age at diagnosis was 8.9 years (14 month to 15 years). There were an equal distribution of genders. Mean duration of fever at the time of diagnosis was 22.4 days. At the presentation all 12 children had fever longer than 2 weeks (100%), 8 children had salmon rash (66%), 9 had arthritis (75%, 100% developed arthritis in the first year of disease), 8 had splenomegaly (66%), only 2 hepatomegaly (16%), 10 children had generalised lymphadenopathy (83%), 5 had serositis (40%). Mild anaemia was present in 75% of patients, leucocytosis in 92%, thrombocytosis in 58%. Elevated sedimentation rate and C reactive protein (CRP) were found in 100% patient. Antinuclear antibody (ANA) was positive in 4 patients (30%).

## Conclusion

Data from our study were consistent with clinical and laboratory findings from other studies and related literature. ANA was positive in 4 children (30%), which is probably false positive result.

## Disclosure of interest

None declared.

